# Under Pressure: Gifted Students’ Vulnerabilities, Stressors, and Coping Mechanisms Within a High Achieving High School

**DOI:** 10.3390/bs15020235

**Published:** 2025-02-18

**Authors:** Addison Helsper, Lillian DeShon, Laura E. Boylan, James Galliher, Lisa DaVia Rubenstein

**Affiliations:** 1 Department of Educational Psychology, Ball State University, Muncie, IN 47306, USA; addison.helsper@bsu.edu (A.H.); llrogers2@bsu.edu (L.D.); james.galliher@bsu.edu (J.G.); 2 Department of Psychology, Virginia Commonwealth University, Richmond, VA 23284, USA; laura.williams1@vcuhealth.org

**Keywords:** gifted, stressors, coping mechanisms, identity development, academics, social, problem-focused coping, emotion-focused coping

## Abstract

This qualitative study explores the interactions among gifted students’ vulnerabilities, stressors, supports, and coping mechanisms at a public, high-achieving residential high school. Qualitative interviews (*n* = 12) revealed that teachers caused stress by (a) failing to provide academic challenges and/or (b) failing to support students’ diverse identities; conversely, teachers provided support when they were available, enthusiastic, and understanding. Further, peers added stress through class rank competition but fostered support through accepting diverse identities and building friendships. In general, students heavily relied on problem-focused coping to address academic stress (e.g., changing schools, advocacy), yet had minimal adaptative coping strategies to address social stress. Students also discussed hybrid coping approaches, like extracurriculars and counseling. These findings suggest that interventions, such as teacher training and peer support programs, should address specific vulnerabilities, including diverse identities, and provide direct instruction in developing coping strategies to address social stressors.

## 1. Introduction

Adolescence is a critical period for developing social functioning, emotion regulation, and higher-order cognitive processes. Exposure to stress during this sensitive time can disrupt development, contributing to an increased risk of psychological disorders (e.g., anxiety, depression) and the reduced plasticity of key brain regions, with effects that may persist into adulthood (Eiland & Romeo, 2013; Sisk & Gee, 2022). This underscores the importance of identifying factors that contribute to adolescents’ stress levels and understanding the coping resources available to them.

The Phenomenological Variance of Ecological Systems Theory (PVEST; Spencer et al., 1997) provides a valuable framework for exploring these dynamics by situating stressors and coping responses within the larger cultural context. PVEST highlights how deviations from the majority (e.g., racial minorities, LGBTQ+ identities, disabilities) create unique stressors and supports that impact students’ coping mechanisms, as well as critical academic and social outcomes.

Giftedness is one such variation that introduces distinct stressors and coping needs, as gifted students are a unique subset with specialized educational needs that may or may not be supported by their surrounding culture. Multiple definitions of giftedness exist; however, within this work, we used the following definition: “students with gifts and talents perform—or have the capability to perform—at higher levels compared to others of the same age, experience, and environment in one or more domains” (United States’ National Association for Gifted Children Definition Task Force, 2018). Although the impact of giftedness on mental well-being relative to peers remains debated (Rommelse et al., 2017; Tasca et al., 2024), research indicates that gifted students experience stress due to the interaction between their personal characteristics and the school environment (e.g., Cross et al., 2015; Hutcheson & Tieso, 2014; Thai et al., 2020), a challenge that is especially pronounced in high-achieving schools (Luthar et al., 2020).

Recently, students in high-achieving schools have been classified as an “at-risk group” due to increased adjustment difficulties, distress, and psychological disorders (Luthar & Kumar, 2018; Luthar et al., 2020; National Academies of Sciences, Engineering, and Medicine [NASEM], 2019). This is during a time when general anxiety, depression, and suicide rates continue to rise (Ehlman et al., 2022; Lebrun-Harris et al., 2022). Further, social media is ubiquitous in many students’ lives, additionally contributing to their stress and anxiety (Keles et al., 2020; Shannon et al., 2022). Given the lasting impact of adolescent stress, it is crucial to examine the unique stressors, supports, and coping mechanisms of gifted adolescents to guide the development of targeted academic and psychological services. Therefore, this qualitative study aims to explore how the unique vulnerabilities of gifted students position them to face specific academic and social stressors.

### 1.1. Phenomenological Variance of Ecological Systems Theory (PVEST)

We anchored this examination on PVEST, a robust theoretical model demonstrating how different lived experiences and identities influence students’ lives (Spencer et al., 1997). Taking inspiration from Bronfenbrenner’s (1979) Ecological Systems Theory, PVEST acknowledges the range of social influence from close family and friends to cultural value systems. PVEST, however, goes beyond, emphasizing individuals’ unique interactions with these systems. Specifically, the “phenomenological variance” communicates how lived experiences vary based on the interaction of personal characteristics within ecological systems. Acknowledging these differences helps to illuminate specific stressors and supports that influence important outcomes and students’ emergent identities.

In an applied iteration, English-Clarke et al. (2012) emphasized these five components: vulnerabilities, stressors, coping processes, emergent identities, and coping outcomes. Within each of these components, PVEST acknowledges the dialectical nature of being a member of an “at-risk” group with unique struggles as well as in-group support systems. Within the current study, we explore these vulnerabilities, stressors, and coping processes within gifted students.

Originally, PVEST was developed to illustrate the significant risks and strong community support associated with being African American (Spencer et al., 1997; English-Clarke et al., 2012); however, the model provides a solid theoretical foundation to explore a variety of vulnerabilities, such as levels of SES, (dis)abilities, and/or LGBTQ status. This is not meant to communicate that all vulnerabilities are equal in influence, but rather, multiple vulnerabilities have unique risks/protective factors, cause stress, and affect development.

#### 1.1.1. Vulnerabilities

Although giftedness may be conceptualized as a vulnerability in and of itself, many gifted students have additional vulnerabilities that interact, such as identifying as LGBTQ (Hutcheson & Tieso, 2014; Lo et al., 2022), being diagnosed with additional exceptionalities (e.g., ADHD; Fugate & Gentry, 2016), and/or being a member of under-represented culture (Plucker, 1998). These combinatory vulnerabilities affect students’ coping responses and, in turn, affect their academic performance, social relationships, and well-being. For example, gifted LGBTQ students need to develop coping mechanisms to face homophobic social environments, which in turn affects their psychological well-being (Hutcheson & Tieso, 2014).

Twice-exceptional students are also uniquely vulnerable as they are not only gifted but also have an additional exceptionality, such as a learning disability or being on the spectrum. Their lived experiences have been examined across contexts, and, collectively, they face a variety of stigma narratives (Ronksley-Pavia et al., 2019), challenges associated with finding appropriate educational placements (Rubenstein et al., 2015), and difficulties with peers and teachers (Abraham, 2025). These vulnerabilities place an additional cognitive load on gifted students as they need to consider how their giftedness interacts with their environment and what coping strategies are necessary to build social capital within specific contexts (Cross et al., 2019).

#### 1.1.2. Stressors/Supports

Collectively, these environmental interactions create stressors and supports for students. Importantly, the same environment might promote both stress and support for a student. Studies within the gifted field have examined different stressors/supports, like participation in an Intellectual Baccalaureate (IB) program (Shaunessy & Suldo, 2009), attendance in a residential, gifted high school (Dixon et al., 2004), and relationships with parents (Mofield et al., 2016). Most studies isolated a specific stressor/support to explore its effects on gifted learners’ coping strategies, examining it through either an academic or social lens. Across those lenses, gifted students experience a wide range of stressors. For example, within the academic lens, students may find stress/support within their teachers (Siegle et al., 2014), and within the social lens, gifted students may find stress/support within their peers (Lo et al., 2022; Unal & Sak, 2023).

##### Academic Stressors/Supports

More specifically, teachers play a dual role in both causing and alleviating stress for gifted students. While they can offer support and encouragement, they may also act on inaccurate assumptions about gifted students, such as the belief that giftedness correlates with poor social, emotional, or behavioral skills (Matheis et al., 2017). These misconceptions can lead to limited support or harsher responses; however, teachers with specific training in gifted education tend to provide more effective encouragement and understanding (Matheis et al., 2017). In addition to teachers’ beliefs about giftedness, teachers also cause stress and provide support through the quality of the academic experiences they provide. This can be caused by too much academic challenge or not enough (Peters, 2022; Riegel & Behrens, 2022; Smedsrud et al., 2022), potentially resulting in a lack of academic growth (Adelson et al., 2012; Redding & Grissom, 2021).

##### Social Stressors/Supports

Gifted students also face significant social stress as they navigate the complex dynamics between gifted and non-gifted peers. This social tension stems not only from how gifted students perceive themselves but also from how others view them (Cross et al., 2019). Non-gifted peers may hold preconceived notions about giftedness, which can lead to unflattering labels like “weirdos”, “know-it-alls”, or “nerds” (Coleman & Cross, 2014; Martínez-Monteagudo et al., 2023). These labels often arise from a misinterpretation of gifted students’ enthusiasm for learning, which can be perceived as arrogance or superiority (Cross et al., 2019). Unfortunately, this social dynamic can sometimes escalate into more serious forms of bullying (Klimecká, 2023). Traits commonly associated with giftedness, such as perfectionism, sensitivity, individualism, and social withdrawal, can make gifted students more vulnerable to negative attention from their peers (Coleman & Cross, 2014; Estell et al., 2008; Martínez-Monteagudo et al., 2023).

These social challenges can be further complicated by the difficulties gifted students face in forming friendships. While spending time with like-minded peers is a crucial coping strategy, many gifted students struggle to connect with non-gifted peers due to differences in interests and social activities (Godor et al., 2020). Friendships typically form around shared hobbies, classes, or extracurricular activities, but the unique academic interests and passions of gifted students may create barriers to building these connections. This lack of meaningful friendships can exacerbate feelings of isolation and stigmatization, leaving many gifted students feeling disconnected from their peer groups (Eddles-Hirsch et al., 2012; Akgül & Kaya, 2023).

#### 1.1.3. Coping Mechanisms

Both academic and social stressors require coping responses. Coping is the behavioral, cognitive, and conscious aim to handle stressful situations either through the modification of the situation or their own response (Folkman et al., 1986). Although there have been many approaches to understanding these responses (e.g., see Skinner et al., 2003 for a review of over 100 assessments and 400 ways of coping), one common approach is by classifying the coping response as either (1) problem-focused coping (i.e., conscious aims to change the stressor at hand) and (2) emotion-focused coping (i.e., conscious aims to change one’s own internal reaction to the stressor; Lazarus & Folkman, 1984). In other words, students respond to stressors by addressing the stressor and/or regulating their responses (Stanisławski, 2019). Depending upon the stressor, either approach could be adaptive, but understanding how and why to employ each type of stressor can lead to more positive outcomes (Folkman & Moskowitz, 2000).

Research within the gifted field has frequently examined coping mechanisms within academic *or* social spheres. First, within the academic sphere, most studies used a quantitative framework to assess academic-focused coping strategies in gifted students, such as the Self-Report Coping Scales (Mofield et al., 2016) and Coping Strategies Inventory (Thai et al., 2020). Gifted students frequently employed problem-solving strategies, wishful thinking, and cognitive restructuring, yet when students used disengagement strategies, they also tended to socially withdraw, whereas those who used engagement strategies had better relationships with their teachers and peers (Thai et al., 2020).

Qualitative results complement these findings by demonstrating how successful gifted students employ specific coping strategies to promote academic success, such as using effective self-regulatory strategies and seeking social support (Siegle et al., 2014; Wu, 2016). One key finding was that students who were most effective at positively transforming their academic performance were students who acknowledged both their giftedness and their underachievement. This demonstrates the power of metacognitive awareness, which is required for developing consistent and systematic coping strategies (Cavilla, 2017).

Next, within the social sphere, the most common social coping scale for gifted students is the Social Coping Questionnaire (Cross & Swiatek, 2009; Swiatek, 2001; Rudasill et al., 2007). Generally, gifted students cope with social stress by denying their giftedness, maintaining high levels of activities, and helping others (Cross et al., 2019; Cross & Swiatek, 2009; Rudasill et al., 2007). Within qualitative work, specifically, gifted LGBTQ students used distinct social coping strategies, including finding supportive friends, hiding their LGBTQ identity, confiding in teachers, and developing their talents (Hutcheson & Tieso, 2014; Peterson, 2000).

### 1.2. Purpose

Although much work has examined gifted students’ vulnerabilities, stressors, and coping responses, they tend to explore them in isolation. They examine twice-exceptionality *or* LGBTQ identities. They explore academic *or* social stressors. Collectively, they examine a single vulnerability and/or specific stressor and coping mechanism, rather than conceptualizing the gifted student as a whole. Therefore, the purpose of this basic interpretive qualitative study (Merriam & Tisdell, 2015) was to holistically examine the academic and social stressors and supports, as well as coping mechanisms, of gifted students within a top-performing public, residential, gifted high school setting. The basic interpretive qualitative methodology is appropriate for this qualitative study, as it seeks to understand and find meaning from the participants but does not include the additional dimensions necessary to be considered a more specific methodology (e.g., phenomenology, grounded theory, ethnography).

The current study also adds to the literature by integrating a new theoretical framework to explore these constructs within the gifted field. Grounded in the Phenomenological Variance of Ecological Systems Theory (PVEST), this study seeks to explore how gifted students’ unique combination of vulnerabilities manifest within their academic and social environments and contribute to their stress levels and the coping strategies they employ. Few studies in the gifted field have anchored their work within PVEST, despite the helpful organizational structure and unique emphasis on both supports and stressors.

Collectively, there is a limited understanding of how gifted students’ unique vulnerabilities—such as their giftedness, intersecting identities (e.g., LGBTQ, ADHD, under-represented racial groups), and school environments—influence their stress levels, coping mechanisms, and developmental outcomes in a *holistic* manner. This study fills this gap in the literature through the use of PVEST as an organizational structure to frame the interactions between environmental and personal characteristics. PVEST acknowledges how dialectal tensions exist across a variety of vulnerabilities, allowing for the comprehensive exploration of how personal and contextual factors interplay, shaping gifted adolescents’ unique stress and coping responses.

To address these gaps, the current study examined both *specific* experiences and *broader* relationships across stressors/supports and coping mechanisms. This study also took a more inclusive approach to the existing coping studies in the gifted field by considering both academic and social stressors and the corresponding coping mechanisms. This work was guided by two research questions:

RQ1: What types of stressors and supports do gifted students experience within their environment?
RQ2: What coping mechanisms do gifted students employ in these stressful situations?

By understanding the specific stressors and supports that affect gifted students, as well as the coping responses that influence their academic and social outcomes, this study aims to provide insights that can inform targeted academic and psychological interventions to enhance their well-being and academic performance.

## 2. Materials and Methods

### 2.1. Participants

The participating students came from a variety of local schools, but all were admitted to the public, residential, gifted high school through a rigorous, multi-dimensional application process, which included assessments of past academic performance, school personnel recommendations, standardized test scores, and essays. Both cognitive and non-cognitive factors were considered by the admissions committee. The high school ranks as the No. 1 College Prep Public High School (Niche, 2025) and provides a liberal art, collegiate-style education with a range of AP, dual credit, and college courses. Highly qualified content experts teach these courses, as all the faculty members have master’s degrees, and one-third hold PhDs in their field of expertise.

This study was a part of a larger study exploring how twice-exceptional students navigate life in a public, residential, gifted high school; however, the sample included both twice-exceptional and non-twice-exceptional students. The researchers and school administrators developed a partnership to recruit participants. The university IRB approved all the procedures (IRB #1955897-1) but also mandated parental permission even though the school is a residential high school, which likely influenced the sample size. Parents/caregivers were contacted via multiple emails and were handed physical flyers as they picked up their students before holidays, which yielded 46 parents/caregivers providing consent. Those 46 students were invited to take the survey during a special class period, and 44 of them gave their assent. Of those 44 students, 12 of them were willing to participate in future interviews. The qualitative participants’ demographic information closely mirrored the initial survey participants’ information, and Table 1 presents more specific information regarding each of the interviewees.

### 2.2. Procedures

This article is a part of a larger mixed-methods study. Phase 1 included an internet survey administered to the students at a public, residential school for the gifted, and Phase 2 involved follow-up interviews with all the participants who indicated willingness. This article only reports on findings from Phase 2. Specifically, the students who agreed to participate in the follow-up interviews were contacted using the information they provided at the end of the survey. They were sent additional information, including an assent form for those under 18 or a consent form for those over 18. Once assent/consent was obtained, interviews were scheduled at mutually agreed-upon times and conducted in person. Each interview was scheduled for 60 min, though the participants could end the interview or skip any questions at any time. The interviews were conducted using a semi-structured interview protocol, found in Appendix A. The interviews were recorded and transcribed.

### 2.3. Data Analysis Plan

To analyze the qualitative data, all the recordings were transcribed and entered into Excel. Each talk turn (i.e., every time the speaker shifted) was placed in its own cell. Then, four authors read and summarized the same two transcripts. Each author tested a different coding approach (Saldaña, 2021) to determine which would be most useful for this data set. Specifically, we explored

- Process codes: identifies the actions/behaviors of the participants.
- Emotion codes: identifies the emotions embedded within the participant’s experiences.
- Conflict (or versus) codes: identifies competing forces.
- Concept codes: provides a bigger picture, assigns a macro or abstract concept to describe the statement.

Within those codes, we saw that the conflict codes captured both the concepts and the emotion codes. For example

“I think [neurodivergence] is more accepted at the [GT School]… I know kids in middle school who have ADHD, and people accept ADHD more than they accept other stuff, but there’s always the stereotype of like “oh, an ADHD kid is just like a rambunctious little kid”. And like, I’m not rambunctious, but they like, label you a troublemaker, but they don’t label you as dumb. They don’t label us mentally ill, but like, when you have like OCD or something, then there’s like, there’s something wrong with you. You know? And I think that’s, that’s part of the difference. Here [at the GT School] things are less stigmatized.”

The coding team identified one overarching conflict surrounding the perceptions of neurodivergence: “stigmatizing and stereotyping v. nuanced complexity”. There is also a conflict between how students are perceived/supported in their “local school environment v. the [GT School]”. Within those conflicts, the participant has emotions, like they feel more accepted, but the participant did not truly discuss their own behaviors. The concept code could be “Trapped in a Label”. Out of these four options, the conflict codes encapsulated both emotions and concepts, whereas process codes did not capture much in this quote or throughout our pilot coding process.

Through these conflict code discussions, we saw significant environmental (e.g., local school vs. [the GT School]) and internalized conflicts (e.g., stigma/stereotype vs. nuanced complexity), which we connected to the dialectical nature of PVEST. We saw supports and stressors, adaptive and maladaptive coping, and positive and negative outcomes. Because those conflicts are inherent within PVEST, we decided to anchor our next round of coding using a structural coding approach with PVEST. Two coders applied this framework to each transcript, met to compare codes, and reached a consensus if they disagreed. After the transcripts were coded using PVEST, the final round of coding used a tabletop coding approach (Saldaña, 2021) to group the examples of each of the PVEST components.

During this process, we implemented Merriam and Tisdell’s (2015) delineated strategies to ensure both validity and reliability. Specifically, collaboration was central to both data collection and analysis, involving multiple investigators at each stage, which enhances credibility by reducing the individual bias and promoting diverse perspectives in interpreting the data. It increases rigor by allowing multiple investigators to cross-check and validate the findings, fostering a more thorough analysis. Additionally, it supports reflexivity and triangulation, ensuring that research is more balanced, inclusive, and trustworthy. We purposefully included individuals with different academic specialties to ensure a more nuanced analysis. We used an audit trail to record our discussions, and further, we anchored our conclusions in rich, detailed descriptions, incorporating full quotes before progressing to broader synthesis. Finally, we practiced personal reflexivity by developing positionality statements that acknowledged how our personal experiences and beliefs may have shaped our interpretation of the data, presented in Appendix B.

## 3. Results

**Figure 1:**
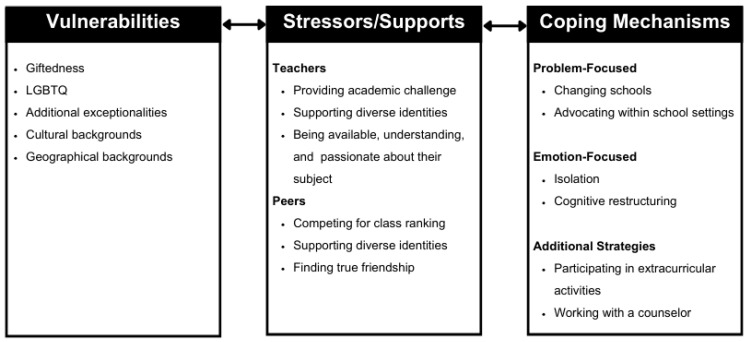
Thematic results for research questions.

Figure 1 presents the results across the research questions, organized through key components of PVEST. Specifically, the participants shared specific vulnerabilities (i.e., giftedness, LGBTQ, additional exceptionalities, and cultural and geographic backgrounds) that brought specific stressors and supports from teachers and peers, which the participants addressed using problem- and emotion-focused coping responses. All of these are supported through the participant quotes in the sections below.

### 3.1. RQ1: How Do Students’ Vulnerabilities Interact with the Stressors and Supports Within Their Environment?

Both stressors and supports arise from the interaction between students’ vulnerabilities and their environment. We defined stressors as emotional or physical tensions that affect students’ behaviors and cognitive processes; conversely, these stressors can often be reduced through meaningful supports. In general, students recognized stressors within both academic and social environments, as delineated below.

Finding #1a--Gifted students experienced a myriad of academic stressors/supports, as evidenced by their experiences with their teachers.

First, teachers play a crucial role in shaping students’ academic experiences, often acting as both sources of stress and support. As explored below, certain teachers may unintentionally contribute to academic and emotional stress by failing to provide adequate challenges, disregarding necessary accommodations, or allowing personal biases to influence their treatment of students. These stressors can result in frustration, disengagement, and a lack of academic progress. Conversely, there are teachers who alleviate stress by being available, understanding, and passionate. These educators create supportive environments that foster student growth, allowing students to feel valued and motivated to succeed. The dual impact of teachers as both stress-inducing and stress-relieving agents underscores their central role in students’ overall well-being and academic success.

#### 3.1.1. Providing Academic Challenge

Many students expressed frustration with the lack of academic challenge in their previous classes, noting how it impacted both their motivation and preparation for future academics. Without adequate challenges, students often felt disengaged or unprepared. Jaime remarked on the similarity between advanced and regular classes in their local school, saying, “They were teaching the same class. Just giving you more work. Same with our English class…you’re reading the same book; you just have to write twice as long as the paper over it. Everyone was kind of miserable together”. This lack of distinction between classes left Jaime feeling academically uninspired, admitting, “It’s kind of the reason I don’t want to go to college”.

Although not every student responded as strongly, many shared negative experiences, often disengaging from their work or feeling unprepared for their future. Charlie mentioned that the absence of meaningful challenges made schoolwork feel purposeless and, thus, harder to complete, and Morgan noted

“People who are considered gifted tend…to get left behind when it comes to like academic support. This was definitely prevalent at my home school. We weren’t really taught how to study or how to learn, so it felt like we hit a brick wall, when it came to learning something [new].”

Reese echoed these sentiments, describing the monotony of unchallenging assignments at their local schools as “busy work…just grinding through information…doing simple assignments over and over”. However, Reese noticed a marked contrast at the GT School, which offered increased rigor and opportunities. They shared,

“Academically, I would say the [GT School] gives you a lot of opportunities to do stuff like research studies, like under college professors, taking college courses…that’s like a motivational factor to be able to get ahead and do more stuff while you can.”

#### 3.1.2. Reacting to Student Differences

In addition to providing (or not) an academic challenge, teachers caused stress by demonstrating significant biases towards students’ other vulnerabilities. Additional stressors included examples in which teachers did not support the students’ differences across LGBTQ identities, cultural backgrounds, and additional exceptionalities. First, Taylor shared how their LGBTQ identity affected interactions with certain teachers, noting that the town’s conservative culture influenced teacher behavior and classroom dynamics:

“[Local Town Name] is a great town, and there’s a wide variety of people. But they’re stuck. They’re still definitely stuck in the 19th century, a little bit. There are a few forward-thinking teachers, and they made the school feel safe. But if you go in the wrong hallway… slurs are thrown everywhere…Sometimes teachers would give you a hard time if you wanted to retake a test, because they knew things about you that they didn’t like. A lot of religion was pushed because it’s such a small town.”

Beyond LGBTQ identities, many witnessed and experienced stressors associated with cultural differences. Cameron discussed the impact of teachers’ nationalistic views on international students, highlighting how these biases could make certain students feel alienated:

“Some of the history teachers…are very pro America. And there are a lot of international students here. So in history class, we will discuss America, like ‘we beat the Spaniards in the war. We are so much better than them…’ And then, my friend from Spain said, ‘This man will not shut up’.”

Further, Charlie recounted an instance where a teacher misunderstood Charlie’s friend’s cultural attire as inappropriate, highlighting the cultural insensitivity that can emerge in educational settings: “they wore an authentic tribal headband with a feather, and their teacher told them to take it off, because ‘it’s a costume’”.

In addition to LGBTQ and cultural identities, some teachers inadvertently increased student stress by disregarding or mishandling accommodations for neurodiverse students. For example, Jordan shared how some teachers failed to respect accommodations outlined in 504 plans, such as giving advance notice for tests:

“I’ve been lucky with the teachers I’ve had…But there are plenty of ones that do pop quizzes. And a lot of kids are like, ‘you can’t just do that to me, [I need] 24 h in advance, I need to set this up and have it in the testing room.’ And some other teachers… They’ll try and mark you absent… But it’s supposed to be excused.”

#### 3.1.3. Being Available, Understanding, and Passionate About Their Subjects

Despite the challenges posed by some teachers, the students also spoke highly of those who provided significant stress relief by being accessible, understanding, and passionate. Although the students reported these types of supportive teachers everywhere, they spoke most highly of their GT School teachers.

First, the stress-relieving teachers were accessible to the students, both to provide academic support and academic opportunities beyond the traditional curriculum. As Reese described,

“I would say the teachers here [at the GT School] are great. Almost all the teachers that I have, they’re always available to reach out to for help, like, anytime you ever need, whereas at my old school, they didn’t have things like office hours. So, if you wanted help, you would have to email them…I never did.”

The supportive teachers were also proactive in their attempts to build relationships with the students. They made themselves available for smaller, directed studies. Dakota described their experiences in this environment, “It’s one of my favorite classes. It’s so much fun. We just like talk about a book for like an hour and a half. I just like it so much!”

In addition to being available, the stress-relieving teachers adapted their levels of support based on students’ needs, as Taylor explained: “they’re all very understanding. They’re very sympathetic when needed, but they don’t baby you. They talk to you as a peer until you give them a reason not to, they respect you”. This level of support extended to twice-exceptional students, as Jaime described: “they’re really good, like they want to work with you to get what’s best for you. They do offer really good 504 plans, but even if you don’t go to the administration, you can just talk to your teacher”.

Many students felt comfortable sharing their struggles and needs because they knew the teachers would exhibit this understanding. Casey discussed how most teachers are “pretty good about it [making accommodations] …like no one is ever snarky about accommodations”.

Although this study was not designed to examine the characteristics of highly effective teachers, as the participants described how many of the teachers provided accommodations and support for students, they also discussed the importance of the teachers’ passions. Jaime described the teacher that has inspired them to learn:

“I love that man [a GT School teacher] so much…he’s just so excited. And he talks really fast. And it’s like the perfect rate, but I’m listening. And he wants to be in the class and teaching, like his favorite part of the day is when it’s time to start class. And he cares a lot, and he puts a lot into his students.”

Collectively, the students’ experiences with academic challenges and supports varied significantly, depending on the teachers and environments they encountered. While some faced additional stress due to the lack of a challenge, unaccommodating teachers, and biases, others found relief and motivation through supportive, accessible, and passionate educators.

**Finding #1b--Gifted students experienced a myriad of social stressors/supports, as evidenced by their relationships with their peers.**

Throughout their time in high school, students naturally shift their primary support systems from family to peers, and this study’s participants made that shift earlier than most as they moved into a residential high school setting. This also gives them a distinct perspective from which to contrast their local peer groups with their GT School peer group.

#### 3.1.4. Competing with Peers

The students highlighted how peer relationships were influenced by the competitive nature of their local schools, where policies (e.g., class rankings) played a significant role in shaping peer interactions. Although these topics were not part of the interview protocol, many students discussed how these systems created stress and competition among peers. At their local schools, the class ranking system fostered a sense of rivalry, as Cameron described:

“[Class ranking] was a big thing. Like this one kid. He didn’t tell anyone else what he was doing. But he like took physics instead of history in freshman year, and everyone else was like, ‘Yeah, we’re gonna take history freshman year to get it out of the way.’ But physics was weighted, and he took physics freshman year. And so he was like, the first, and there were like 30 people tied for second place.”

At the GT School, however, the absence of a ranking system significantly reduced this type of competition, which changed the dynamic among students, and Cameron noted the contrast, “Well, we don’t do class ranking, so…it’s kind of funny that it’s less competitive here than it was in my old school”.

Other students shared similar views, expressing that without the pressure of rankings, they felt more comfortable recognizing each other’s strengths. Quinn explained, “Just knowing that everyone around you is smart, and that you could be better or worse than another person, but you just don’t know, especially because everyone has strengths in different locations. I think it’s just good overall”. By removing formal academic hierarchies, the GT School allowed students to focus more on their individual growth and collaboration with peers, rather than viewing each other as rivals in the pursuit of higher rankings.

#### 3.1.5. Supporting Peers’ Diverse Identities

Many students shared that, similar to teachers, their peers, especially in their local schools, often lacked understanding of diverse identities, which created social stress. This was especially true for students identifying as LGBTQ+ or demonstrating neurodivergence. For example, Taylor described the stigma they faced as a member of the LGBTQ community: “If you go in the wrong hallway… slurs are thrown everywhere. But they’re not very creative”.

In addition to the challenges of being LGBTQ+, students also discussed the stress associated with neurodivergence, including both giftedness and other exceptionalities. Dakota noted the stark difference between the local school environment and GT School when it came to the acceptance of neurodivergent individuals: “Back home, definitely, definitely [there’s a stigma surrounding neurodivergence]. Here [at the GT School], not really, because everyone is a little neurodivergent in their own way”.

This contrast was a recurring theme, as many students recognized that their peers at the GT School were more understanding and accepting. The exposure to a diverse peer group at the GT School helped students see their differences as strengths, rather than sources of isolation. Casey reflected on this shift, comparing the social stressors at their local school to the support they found at the GT School:

“I’m trans and… people are annoying. And like, it’s fine. You get used to it, but there’s a lot of LGBT hate, and my old school was also like, completely white. And I came here [to the GT School]… and I met a lot of different people and got more worldview perspectives and stuff. So besides being academically challenged, I’ve met a lot of cool people. And people, even if they don’t like you, like, they’re not going to go up to you and be mean.”

For many students, their time at the GT School also helped them recognize their own gaps in understanding and become more supportive of others. Several students acknowledged that they had not been exposed to the vulnerabilities or diverse experiences of others in their hometowns. This exposure at the GT School helped them grow as individuals and become advocates for their peers. Dakota described how a diversity and inclusivity training at the beginning of the year opened their eyes: “We did a training at the beginning of the year on diversity and inclusivity, which definitely helped because from being in the middle of nowhere, I didn’t know any of this stuff”.

In addition to the general appreciation for diversity, students also began to understand and appreciate neurodiversity. Quinn, who did not have a learning disability, expressed admiration for peers with disabilities who were able to succeed academically despite the challenges:

“Yeah, I found it very impressive that the students that had these learning disabilities were still able to keep up with this high learning curve, which proves that just because you have a learning disability doesn’t necessarily mean that you can’t be one of the top-tier students.”

Without prompting, five different students mentioned how much they valued the exposure to diverse identities at the GT School. This increased understanding not only helped them feel more supported but also encouraged them to provide that same support to others.

#### 3.1.6. Finding Friends

For some students, their local school communities provided opportunities to build close-knit connections through clubs, sports, and other activities. Quinn reflected on the sense of community they had experienced at their former school:

“It’s so small, everything was really close-knit. Especially with sports. I did sports back there too, and I was so sad that I had to leave my sports team. And I also feel like it was getting better because [my town] really cracked down on bullying.”

While several students expressed sadness about leaving their local schools and peers behind, many more found that the GT School offered the chance to form deeper, more supportive friendships. At the GT School, students connected with others who shared their academic interests and personal experiences. Charlie explained how much easier it was to make friends at the GT School compared to their previous school:

“I never really was able to make friends [at the local school] because I thought they were just loud and obnoxious. But yeah, here [at the GT School], it feels really easy to make friends, which I found very surprising.”

These connections often revolved around shared passions and intellectual pursuits. Dakota described how their friendships at the GT School were built on common interests and mutual understanding: “There are more people that I feel like I have a deeper connection with… like on the same level, people that I can empathize with, that are going through the same thing I am”.

However, the similarity between students sometimes caused unintended stress. Cameron shared how having friends who dealt with similar struggles, such as eating disorders, could lead to challenges:

“With my little eating disorder thing, there’s like a lot of people at the school who also have an eating disorder. Like when I first met my friends, I didn’t know any of them did, but four out of the five people in my friend group have an eating disorder. So sometimes, we accidentally trigger each other just by talking about our life experiences.”

Despite these challenges, all the students expressed feeling supported by their peers at the GT School. Taylor described the community as a place where students, even if not best friends, were still kind and supportive: “It’s a very tight-knit community, even if you’re not best friends with everyone. And everyone’s friendly and decent”.

Reese echoed this sentiment, emphasizing the acceptance and understanding within the GT School community: “Peers have been great. I feel like most of the people here are accepting and understanding, which is great for making new friends”. This sense of community and shared understanding at GT School fostered friendships that were both emotionally supportive and academically enriching. Ultimately, these peer connections played a crucial role in helping students navigate the challenges of transitioning to a new environment while feeling valued and accepted.

### 3.2. RQ2: What Coping Mechanisms Do Gifted Students Employ in Stressful Situations?

Finding #2: Students implemented several problem- and emotion-focused coping strategies; however, most had not developed a systematic/planned approach to coping with stress, especially in the social domain.

The previous finding demonstrated the students’ abilities to describe in-depth their academic and social stressors and supports, which should be the first step in recognizing the need for specific coping strategies and plans. However, when asked about their coping strategies, many (not all) of them described limited approaches, and most of those centered around their academic stressors. In the sections that follow, we chose to code based on problem- and emotion-focused coping strategies.

#### 3.2.1. Problem-Focused Coping

First, problem-focused coping refers to strategies where individuals actively work to address, manage, or resolve the source of their stress or challenge. This approach involves identifying the problem, considering potential solutions, and taking concrete steps to reduce or eliminate the stressor, rather than focusing on emotional responses (or regulating internal responses). The aim is to directly tackle the issue causing the stress to improve the situation.

##### Changing Schools

First, problem-focused coping depends on directly addressing the problem, and these students primarily emphasized addressing big academic challenges using problem-focused coping strategies. All the students decided to switch schools, as they recognized they were not being academically challenged in their local schools. As Jaime described,

“I don’t really do school very well, the hope with coming to the [GT School] was that I would feel less like I was wasting my time. Because my old school is just like, busy work all the time. And the teachers that didn’t really care to be there. And so then I didn’t really care to be there. My dad thought I’d have dropped out by now. But here we are. We’re still in. I plan on graduating.”

##### Advocating Within School Settings

In addition to switching schools, the students also discussed how they needed to self-advocate across educational settings. Sometimes, they advocated for more challenging opportunities, like being able to take a specialized criminal law course (Taylor), whereas others advocated for additional academic support, like being able to take tests in different locations (Casey). Even within a specialized school for the gifted, students needed to come prepared to advocate for themselves, as Logan explained:

“I know that there are some people who have trouble with their advisors, or, like advisors don’t know, as much about all of the classes that are offered, so they give false information. Or don’t tell a student that they could take a class when they actually could so they miss an opportunity… Mostly, I haven’t had that problem, because I’m a big preparer. I like to feel prepared for any situation… So before, like all of my advisory meetings, I come knowing exactly what I want.”

Collectively, these students used problem-based coping strategies at a macro level to address their academic need for challenge by adjusting (a) the school they attend and (b) the classes they select. However, none of them mentioned advocating for additional challenges at the teacher level, but they did feel comfortable advocating for accommodations as a part of their other exceptionality, like longer time for tests, being excused to a sensory room, or to see the counselor during class. Further, many have developed micro-level strategies to support their study habits, like studying with friends (Morgan), listening to music (Casey and Charlie), developing a personal nightly schedule (Jaime), and regulating their stimuli (Morgan).

When these students discussed their problem-based approaches to coping, their responses centered on addressing their academic stressors. Even when they discussed making new friends, they often selected new friends that would push them to reach their academic goals (Reese, Jaime). In general, social stressors were discussed frequently, but problem-based coping strategies for those social stressors were not. When they did report the use of any strategy for social stress, they seemed to resort to avoidance (e.g., physically avoiding certain hallways in their local schools—Taylor).

#### 3.2.2. Emotion-Focused Coping

In addition to problem-focused coping, students also used emotion-focused coping (i.e., coping that directly influences the individual rather than the problem). Emotion-focused coping is a strategy where individuals manage their emotional responses to a stressor, rather than trying to change the stressor itself. The goal is to alleviate the emotional impact of the stressor, especially when the source of stress cannot be directly altered.

##### Isolation

The most popular example of emotion-focused coping was isolation and avoidance (Alex, Taylor, Casey, Quinn, Dakota, and Jaime). Both Alex and Jaime described how they used to employ this strategy, but have since tried to avoid it, yet they did not replace the strategy with a specific new approach. Jaime described how they “find it really easy to isolate myself and sit in my bedroom all day by myself. Um, it was to the point where like, people were having to drag me out of my room”.

##### Cognitive Restructuring

Another popular example of emotion-focused coping within our sample was cognitive restructuring, during which the students noticed and reframed their own thinking patterns. Within these interviews, the students demonstrated restructuring without necessarily being fully aware that they were doing so. For example, students restructured their initial thoughts around whether “everything” was their fault or responsibility (Taylor) and whether they could find comfort in the struggle (Morgan).

The most popular example of cognitive restructuring, however, was when they described their own giftedness (e.g., Alex, Quinn, and Dakota). Specifically, in the beginning of the interviews, we discussed the language that would be used throughout the interview to ensure the participants felt at ease, including the language surrounding gender, giftedness, and exceptionalities. While the students were able to address most of these questions with ease, the students tended to struggle to use the word “gifted” to describe themselves. They provided multiple caveats, like they are simply motivated, they work hard, or they enjoy learning. Alex explained the dilemma: “I don’t want to go around saying it too much. I don’t want people to think of me as on a high horse or whatever”.

##### Participating in Extracurricular Activities

Finally, another popular emotion-focused coping strategy was their participation in a wide variety of clubs, sports (Logan), and other activities. The activities included both traditional and more personalized activities. For example, the students described hanging out with friends (Taylor and Logan), listening to music (Casey), being outside (Taylor and Cameron), and playing sports (Alex, Logan, and Reese), but they also described new clubs that they proposed and were sanctioned by the school, including, humorously, cat club, whose primary activity is looking at pictures of cats, and, more seriously, the Student Equity Council. The council’s goal is to teach everyone about equity through various programs and to bring awareness to incident reports (Cameron).

#### 3.2.3. Beyond Problem- and Emotion-Focused Coping

Some coping strategies were challenging to code as either problem- or emotion-focused coping because they could include both (e.g., counselling), and some students described employing minimal coping strategies.

##### Counselling Opportunities

Therapy and various treatment options could be both problem- and emotion-focused if the professional teaches the student how to regulate their emotions *and* how to solve specific problems. Several participants described their experiences with therapy and the various strategies and treatments they encountered. Logan shared that their counseling sessions were primarily aimed at eliminating their ineffective coping mechanisms but ultimately led them to develop greater self-respect and an understanding of their mental processes; they noted, “I was having a better time dealing with things once I could figure out kind of how my brain was working. Like the scientific reasons behind things made you feel like less overwhelmed”. Morgan recounted receiving a formal diagnosis, beginning treatment with their current psychiatrist, and being prescribed various medications. They mentioned that they are now in therapy with the [GT School] therapist and are generally doing well. Further, Charlie recalled an early experience with a therapist who introduced them to a grounding technique involving the five senses, which they found helpful in managing anxiety.

##### Lack of Strategies

While these students had opportunities to work with professionals, many of the students demonstrated a limited awareness or utilization of specific coping strategies. Some, like Alex, explained that stress is their motivator, such that they do not need additional strategies if they are stressed. Beyond simply appreciating stress for its motivation, many of the students described situations in which they manage stressors without consciously employing coping mechanisms, as exemplified by Alex’s statement: “I’ve always made it without them. I mean, there might be some I use that I don’t realize, but I’ve never actively, like, go and trying to do that”. Similarly, Reese explained, “I don’t really have any coping strategies. Because I am not really stressed out? Yeah, I can just manage as we go”.

However, some realized that this approach may not work in future college or career settings. Dakota stated,

“Um, I know I spend a lot of time on homework, which I never had to do. So I have less free time. And classes are harder so I have to study for them actually, which is also a new. Then the only challenging thing would be like study skills. I don’t have study skills. So I have to somehow find a way to do that. And also make time to actually do other things other than homework.”

Morgan corroborated the challenge of coping when they have minimal strategies at their disposal with an analogy: “if you have one hammer that breaks through every wall, when you get to that wall that you can’t break, you don’t really know what else to do, because you’ve just been smashing every wall with the hammer”. These students’ situations highlight the potential consequences of not having established coping strategies when confronted with increased academic rigor. Overall, these accounts reflect a range of experiences from the students, most of whom have not actively cultivated a range of coping strategies, either due to the perceived lack of necessity or unfamiliarity with such techniques.

## 4. Discussion

### 4.1. Key Finding #1: Gifted Students Maintain Multiple Complex Identities

From our demographic data, we identified that gifted students often possess not just one additional vulnerability but multiple, such as being a member of the LGBTQ community while also having another exceptionality, like ADHD or a learning disability. These intersecting identities interact with their environment, creating unique stressors and supports. For instance, addressing students’ academic giftedness may provide them with appropriate intellectual challenges, but this does not ensure that their LGBTQ identity is recognized or supported. When one aspect of their identity is nurtured but another is dismissed or marginalized, it can lead to significant emotional and psychological stress, ultimately affecting both academic performance and social relationships. This underscores the need for a more holistic approach to supporting gifted students, one that acknowledges the full spectrum of their identities and vulnerabilities.

For example, a gifted student who excels academically but faces homophobic environments may experience decreased self-esteem or social isolation, which can detract from their overall success in both school and personal development. Simply understanding this sample’s demographic composition is a reminder that gifted students maintain multiple identities in the classroom and research should recognize those complex identities.

### 4.2. Key Finding #2: Gifted Students Experience the Dialectal Nature Represented in PVEST

Gifted students have a wide range of vulnerabilities, which causes great stress but also provides opportunities for support. These qualitative findings shed light on *how* students perceived this support (or the lack thereof). Specifically, the students felt supported when teachers provided academic challenges, respected and upheld their diverse identities, and were consistently available and understanding. These results align with earlier research, which found that effective teachers promote student achievement by building strong social relationships, possessing subject expertise, and ensuring an appropriate level of academic challenge (Siegle et al., 2014; Wang & Eccles, 2012).

In addition to teachers, students recognized how peers both cause stress and offer support. For gifted students, who may experience social isolation or struggle to fit in due to their intellectual abilities or unique identities, supportive peer relationships can provide a vital source of emotional stability and acceptance. Peer relationships can be especially stressful as peers compete for class rankings, and some students experienced peer ridicule because of their unique identities. This reflects the existing research indicating that gifted students can feel isolated from their peers, tasked with additional academic responsibilities, and targeted for bullying (Klimecká, 2023). Further, gifted students with additional vulnerabilities can experience additional stressors (Neihart et al., 2002), such as LGBTQ students confronting homophobic environments (Lo et al., 2022). However, peers were also supportive as they expressed true acceptance for multiple identities and built strong friendships. These supportive relationships are also represented in existing research. For example, many LGBTQ adolescents coped with those homophobic environments by finding a small group of supportive friends (Hutcheson & Tieso, 2014).

Our study, however, adds a crucial dimension to the existing literature by emphasizing the importance of acknowledging and supporting students’ multiple, diverse identities, a theme that emerged prominently for both teachers and peers. The students not only recognized when their own identities were dismissed or undermined but were also keenly aware of similar instances affecting their peers. For example, they observed the biases of their history teachers, as they empathized with their international classmates. They also noticed when their peers’ educational accommodations, such as 504 plans, were not appropriately followed. These students placed significant value on the recognition and support of diversity, both within the classroom and in their social circles. Previous research has suggested that gifted students often display heightened empathy (Hay et al., 2007), and our study extends this understanding by demonstrating how this empathy manifests in today’s educational climate and social context.

### 4.3. Key Finding #3: Gifted Students Did Not Demonstrate Equal Levels of Academic and Social Coping Skills

Within our findings, the students tended to discuss problem-based coping mechanisms within academic settings; however, they were not able to discuss similar coping mechanisms for social settings in the same level of detail. Similarly, the important findings are not only what students say they do, but what they fail to mention. These gifted students were able to advocate for accommodations based on their additional exceptionalities, but none of them discussed advocating for more challenging work. In the current study, these students wanted academic challenges/opportunities enough to change schools, yet no one mentioned discussing the lack of challenge with teachers at their local schools. This aligns with previous work, which found that gifted students tend to use behavioral-avoidance strategies during periods of boredom or insufficient challenge, rather than ask for more challenging work (Hinterplattner et al., 2022).

### 4.4. Limitations and Future Research

One initial limitation is the snapshot nature of a single interview. Many factors may confound student responses, such as the timing during the semester or recent peer/teacher interactions. Future work could employ a longitudinal approach to examine how stable students’ responses are across time. This work could use experience sampling to study how students’ stressors and coping responses change throughout the day, week, or even semester. Specifically, students would be contacted at random intervals throughout the study and asked to report their in-the-moment levels of stress, support, and coping. This would allow researchers to determine a more nuanced environmental examination of gifted students’ experiences. At a more macro level, a longitudinal study would be helpful to determine how these various factors support or inhibit students’ broader identity development.

Additionally, the unique nature of this sample may limit the generalizability of the findings to gifted students at large. The students in this study attended a residential, gifted high school where most students lived on campus during active classes, which may not reflect the experiences of gifted or twice-exceptional students in more traditional school settings. Future research should explore whether students in different educational and living environments experience similar stressors and employ similar coping strategies.

Next, students’ language rarely matches the language used within research. Although many students could articulate their emotional states and reactions, fewer could discuss their coping mechanisms, potentially because they do not have the terminology to do so. For example, we recognized students’ using cognitive restructuring, particularly pertaining to their giftedness label, as a coping mechanism, but none of the students were able to recognize that they use a strategic coping response when asked. Without the language and recognition, students may not be able to transfer helpful strategies to new contexts. Therefore, future work may want to intentionally teach different strategies, especially social coping strategies, to provide students with the necessary background information and application practice to crystallize their intentional use.

Future research may also want to test the use of microanalytic interviews to determine how students approach specific situations. For example, a researcher may want to propose a stressful situation and then guide student participants through a series of specific interview questions regarding how they would prepare to cope with the stressor, how they would cope, and then, finally, how they would reflect upon that experience. This would allow for more situation-specific information to better determine the efficacy of their coping response for those specific situations.

## 5. Conclusions

Our study sheds light on the diverse identities and vulnerabilities of gifted students, demonstrating how both academic and social support play a critical role in their well-being. However, the ability of gifted students to navigate these stressors and supports is shaped by both their coping strategies and their settings (i.e., academic vs. social). While the students reported the extensive use of their academic coping strategies, their ability to report or reflect on their social coping strategies was more limited. Specifically, the qualitative findings emphasized that while many students successfully advocated for accommodations related to their exceptionalities, they often hesitated to seek out more challenging academic work, underscoring a gap in self-advocacy. These results highlight the need for a holistic approach to supporting gifted students that not only nurtures their intellectual capabilities but also recognizes and embraces their diverse identities, offering tailored support to help them thrive both academically and socially.

## Figures and Tables

**Table 1:** Qualitative demographics.

ID *	Gender	Race	Sexuality	Exceptionalities
1	Male	White	Straight	
2	Third-Gender	White	Queer	ASD, Learning Disability, Depression.
3	Third-Gender	White	Pansexual	Anxiety **.
4	Third-Gender	White	Queer	Anxiety, Depression.
5	Trans-Woman	White	Demisexual	ADHD, Anxiety, Sensory Processing, Depression.
6	Trans-Man	White	Bisexual	ADHD, Anxiety, Depression, OCD.
7	Female	Multi-Racial	Hetero–Demisexual	
8	Male	White	Straight	Anxiety
9	Female	White	Bisexual	Anxiety, Depression, Depersonalization–Derealization Disorder.
10	Female	AI/AN	Pansexual	Depression.
11	Male	White	Gay	
12	Female	White	Bisexual	Learning Disability.

* All the students were given gender-neutral pseudonyms for this article. This was in an effort to protect the anonymity of the students’ quotes. ** Current diagnosis but feels they have been misdiagnosed for their whole life. Additional Notes. ASD = Autism Spectrum Disorder. ADHD = Attention Deficit Hyperactivity Disorder. OCD = Obsessive Compulsive Disorder. Third-Gender = not clearly viewed as male nor female. Trans-Woman = women who were assigned male at birth. Trans-Man = men who were assigned female at birth. Queer = umbrella term for not having heterosexual “straight” attractions. Pansexual = sexual attraction to all genders. Demisexual = experiences sexual attraction only once they have created a romantic or platonic relationship with them. Bisexual = sexual attraction to more than one gender. Hetero–Demisexual = does not experience primary sexual attraction until bonds are made. Gay = sexual attraction to the same gender as themselves.

## Data Availability

Data are unavailable due to privacy restrictions and ethical concerns.

## Appendix A

Interview Protocol
Hello! I am __(name)__, and I am a ___(educational level)___ student in ___(program)___ at Ball State. I am working with (the PI) to study your experiences before you arrived at the [GT School] and then, more recently, your experiences at the [GT School].

We are excited to hear about all of your experiences, including both the positives and negatives. In general, the purpose of this study is to understand how gifted students with exceptionalities navigate educational environments. Our greatest hope is that these interviews will be used to gain preliminary information to guide future programmatic changes as well as supporting other high schools in implementing effective educational practices. As a reminder, all your responses will remain confidential as I will blind the transcripts as soon as you approve them, and none of your personal information will appear in future publications.

Section 1: Introduction

- First, tell us a little about yourself.
- What are you passionate about? What do you love to do?

Section 2: Establishing Language
Transition: Now, I would like to get to the heart of the interview, but before I do, I want to make sure I am using your preferred language:

- Do you consider yourself gifted? Why or why not?
- Does a better word describe your strengths? [If yes, interviewer needs to use that word.]
- Do you think you have at least one additional exceptionalities, like ADHD, ASD, or a learning disability? [Interviewer: note the interviewee’s language on additional exceptionality to embed in future questions.]
- When did you start to think you had [this additional exceptionality]? How did you know?
- Have you been officially diagnosed? [Follow-ups if yes: By whom? When? How?]

Section 3: Reflecting on Local School Experiences
Transition: Now, let’s think back a few years to examine your experiences at your local school.

- Tell me a little about your local school. [Follow-up: location, values, resources.]
- To what extent do you feel that your home school was able to meet your needs? [Follow-ups: Were they able to academically challenge you? Did they provide you appropriate supports?]
- How connected did you feel to your home school? [Follow-up: Tell me about your closest friend? What about teacher?]
- What types of assignments were you given in your home schools? [Follow-ups: How much time did you spend studying? Do you feel like you developed your competence? Your ability to regulate yourself? Do you feel like you were reaching your potential?]
- To what extent do you think your home school influenced you academically? Socially? Emotionally?
- While you were at your home school, did you establish any coping strategies to help you mentally? Were you taught any coping strategies? [Perhaps, list them.]
- Did you receive an IEP or a 504 plan in your home school? [Follow-ups: What accommodations did you receive? Did you get your accommodations? How did you feel about your accommodations?]
- How are your experiences at the [GT School] different from or similar to your experiences at your home school?

Section 4: Reflecting on the [GT School] Experiences
Transition: Now, let’s focus only on your time at the [GT School].

- What motivated you to attend the [GT School]?
- How has being here impacted your view of “giftedness”?
- Has being here impacted your self-confidence?
- How has your experience here impacted your view of [learning difficulties or neurodivergence]?
- What are some of the challenges you have experienced while attending the GT School? [Follow-up: Tell us about your experience with teachers. What about peers?]
- What is your opinion on how administrators handle working with individuals who have both gifts and disability?
- Do you feel like all your needs are being met at the GT School? [Follow-ups: Do you receive an accommodation at the GT School? Do you have any difficulty in the implementation of your accommodation?]
- What would be an accommodation you feel would better serve you?
- What types of assignments are you given at the [GT School]? [Follow-ups: How much time do you spend studying? Do you feel like you are developing competence? Developing your ability to regulate yourself? Do you feel like you were reaching your potential?]
- How connected did you feel to the GT School? [Follow-up: Tell me about your closest friend? What about a teacher?]
- Do you feel any stigma associated with [neurodivergence or twice-exceptionality]? Do you feel it for yourself or others?
- To what extent do you think the GT School has influenced you academically? Socially? Emotionally? [How much do you study?]
- I know the [GT School] is supposed to provide a rigorous education, so I am curious have you established any coping strategies to help you mentally? Were you taught any coping strategies?
- If you could design the [GT School] to meet the needs of all neurodivergent students, what would be your first initiative? [Follow-ups: Then what?]

Section 5: Conclusions
Transition: You have provided us with such rich information! I just have one last questions for you. It has been a few years since I was in high school, so I am curious:

- What should I have asked you about that I didn’t think of?

Thank you so much for your time.

## Appendix B. Positionality Statements

Author #1 is a white, non-binary researcher raised in a low-earning socioeconomic environment: I bring perspectives and biases shaped by my background in Western, English-speaking culture. My academic training includes an MS in quantitative sciences and a PhD in progress in educational psychology, which have equipped me with a solid foundation in research methodologies. My extensive experience with coping studies, while not specifically focused on gifted students, informs my understanding of the psychological constructs involved. I approached this study without any prior relationships with the participants, ensuring an objective lens in data collection. However, my prior work on models, such as the stress-appraisal model, has influenced my approach to interpreting coping mechanisms in this context. I remained reflexive throughout the research process, acknowledging how my background and theoretical orientations shape the questions I ask and the interpretations I draw.

Author #2 identifies as a white, female researcher raised in a low-earning socioeconomic environment: I bring perspectives and biases shaped by my background in Western, English-speaking culture. My academic experience includes an MS in educational psychology and a PhD in progress in educational psychology, which have equipped me with a solid foundation in research methodologies. Extensive experience in high ability studies and human development has given me a strong foundation. I approached this study without any prior relationships with the participants, ensuring an objective lens in the research process.

Author #3 identifies as a white, female researcher raised in a middle-class socioeconomic environment, who brings perspectives and biases shaped by her background in Western, English-speaking culture. With a BS in family and community services and a PhD in progress in developmental psychology, she has a unique lens for understanding the needs of individuals within systems and across various stages of life. Her extensive experience with emotion regulation studies, while not specifically focused on coping strategies used by gifted students, informs her understanding of the psychological constructs involved. However, her experiences as an early childhood instructor and K-12 emotional disabilities classroom teacher have influenced her approach to interpreting students’ psychological and physical needs in this context. By remaining mindful of these influences, she approached this project with fairness and a commitment to balanced, thoughtful interpretation.

Author #4 identifies as an author with Attention Deficit Disorder (ADD), depression, and anxiety. They acknowledge that their personal experiences shape their perspective on the topics they research and discuss. Growing up in an era when mental health was not widely recognized or addressed, they did not benefit from specialized education or exceptional programs designed to support students with diverse learning needs. This lack of institutional support has influenced their understanding of the challenges faced by individuals with similar conditions. While these experiences undeniably shape their outlook, they strive to approach the data with objectivity and a commitment to unbiased analysis. They remain mindful of the potential for their lived experiences to inform their interpretations, and they make conscious efforts to minimize any favoritism or undue influence from their personal background. By doing so, they aim to ensure that their work reflects a balanced and accurate representation of the data, while also acknowledging the broader social context in which they, and others like them, have navigated their academic and personal journeys.

Author #5 identifies as a white woman. She was not identified as gifted but had the opportunity to participate in honors/AP courses, which were her favorite courses because they were academically challenging. She previously served as a gifted program coordinator and classroom teacher, and currently, as a professor, she teaches courses in gifted education. Through those experiences, she has witnessed multiple stressors and supports that affect gifted students; however, throughout this process, she has worked to center the participants’ own words to inform her analysis.
